# The efficacy of mifepristone-misoprostol regimen versus misoprostol-only for medication abortion at 22 + 0/7 to 30 + 0/7 weeks’ gestation

**DOI:** 10.1007/s00404-024-07737-2

**Published:** 2024-09-18

**Authors:** Or Touval, Avital Ellert, Yair Daykan, Ron Schonman, Zvi Klein, Yael Yagur

**Affiliations:** 1 Department of Obstetrics and Gynecology, Meir Medical Center, 59 Tchernichovsky St, Kfar Saba, Israel; 2 Affiliated with the School of Medicine, Faculty of Medical and Health Sciences, Tel Aviv University, Tel Aviv, Israel

**Keywords:** Mifepristone, Misoprostol, Abortion, Termination of pregnancy

## Abstract

**Objective:**

This study aimed to compare duration of medication abortion after pretreatment with mifepristone versus misoprostol-only regimens at 22 + 0/7 to 30 + 0/7 weeks.

**Methods:**

This retrospective cohort study included patients admitted for medication abortion from 2014 to 2022. Patients underwent feticide due to genetic or anatomical abnormalities at gestational age of 22 + 0/7 to 30 + 0/7 weeks. Excluded from this study were patients admitted at gestational age < 22 + 0/7 or > 30 + 0/7 weeks, with multiple gestation, with diagnosis of intrauterine fetal demise before feticide, with contraindication for vaginal delivery, and who were administered a medical regimen other than the mifepristone-misoprostol or misoprostol-only protocol. Information collected included patients’ demographics, clinical outcomes, additional procedural interventions, and complications. Data of patients treated with mifepristone-misoprostol versus misoprostol-only were compared.

**Results:**

The study group included 46 patients in the mifepristone-misoprostol group and 35 in the misoprostol-only group. Median interval from first dose of misoprostol to fetal expulsion was shorter in the mifepristone-misoprostol group (10.6 vs. 15.3 h; *p* = 0.007) with shorter duration of hospitalization (3.5 ± 1.1 vs. 4.1 ± 1.2 days; *p* = 0.013). Study groups did not differ in terms of complications. Patients in the mifepristone-misoprostol group had a younger gestational age (23.8 ± 1.69 vs. 25.37 ± 2.4 weeks; *p* = 0.002). However, multivariable Cox regression found that mifepristone was independently associated with shorter abortion time (OR 1.7, 95% CI 1.03–2.9, *p* = 0.03).

**Conclusion:**

Medication abortion with mifepristone-misoprostol was associated with shorter time to fetal expulsion at gestational ages 22 + 0/7 to 30 + 0/7 weeks, compared with misoprostol-only regimen.

## What does this study add to the clinical work

**Table:** 

Pretreatment with mifepristone for medication abortion at ≥ 22 + 0/7 to < 30 + 0/7 weeks might reduce abortion interval without additional complications.	

## Introduction

Abortions in the second and third trimester of pregnancy are infrequent when contrasted with first trimester abortions. They arise because of intrauterine fetal demise (IUFD) or genetic and anatomical anomalies. Depending on the local laws [1, 2], termination of pregnancy (TOP) can be achieved medically or surgically, based on patient preference and clinical expertise. In the second trimester (> 13 + 0/7 weeks), the common surgical method is dilatation and evacuation, requiring cervical preparation beforehand and skilled practitioners [1].

Medication abortion is the expulsion of the fetus from the uterus using pharmacological preparations. Misoprostol is a prostaglandin analogue that induces myometrial contractions to expel the fetus. Mifepristone is a competitive progesterone antagonist that primes the myometrium before the use of misoprostol [3].

The use of mifepristone-misoprostol regimen for medication abortion in the second trimester is effective and shortens the interval to fetal expulsion, compared with the use of misoprostol alone [4–10].

Despite available data, some gaps in knowledge persist. Since abortions after gestational age (GA) > 20 + 0/7 weeks are less common, most data are based on patients at earlier GA. In addition, studies that evaluated patients at later GA, included patients ranging from 14 + 0/7 to 28 + 0/7 weeks’ gestation in the same group [4–9, 11]. Other studies that exclusively included patients ≥ 22 + 0/7 weeks’ gestation did not find a clear correlation between mifepristone and a shorter induction interval [11]. However, cases included in these studies were diverse, including IUFD and abortion after feticide in the same group, and relied on protocols that used various additional induction methods [11–17].

Abortions are physically and emotionally stressful events and may lead to short- and long-term complications. Thus, shortening the duration of the procedure can have a significant impact on a patients’ experience. A shorter abortion time can also reduce the total length of hospitalization, alleviate pain, and enhance overall patient satisfaction [8, 18, 19].

Although there is good evidence to support the use of mifepristone for medication abortion in the early second trimester, the data supporting its use at > 22 + 0/7 weeks’ gestation are limited. Recognizing the substantial disparities in the literature and acknowledging the advantage of a reliable and efficacious protocol, this study examined the outcomes of two groups of patients who underwent medication abortion at 22 + 0/7 to 30 + 0/7 weeks. One received the misoprostol-only protocol and the other the combined mifepristone-misoprostol protocol.

## Methods

### Patients

This retrospective cohort study included patients who were admitted to the gynecology unit at our tertiary, university-affiliated medical center, for medication abortion at 22 + 0/7 to 30 + 0/7 weeks, from January 2014 to May 2022. Patients were ages 18–45 years with indications for medication abortion based on medical reasons, or fetal genetic abnormalities or anatomical malformations.

Excluded from the study were patients with multiple gestation, GA < 22 + 0/7 or > 30 + 0/7 weeks, who were administered other medical regimens for abortion (i.e. prostaglandin E2, osmotic dilator or balloon catheter), contraindication for vaginal delivery, > 3 previous lower uterine segment or one previous classical or T-shaped incision for cesarean delivery (CD), IUFD, spontaneous rupture of membranes or onset of labor (before or after feticide), known sensitivity to the medications used, or with hemodynamic instability (due to bleeding or other medical illness) that required urgent intervention for uterine evacuation.

### Departmental protocol

Medication abortion was performed after the approval of the institutional Pregnancy Termination Committee, according to the national Termination of Pregnancy Law. All patients were hospitalized for the procedure. The GA was established based on first trimester transvaginal ultrasound (US) determined by a certified sonographer. To prevent unintended live births, the department’s protocol was to perform feticide prior to medication abortion at 22 + 0/7 weeks’ gestation or later, or when ultrasound (US) estimated fetal weight was > 500 g. Feticide was performed using intra-amniotic injection of digoxin or direct injection of potassium chloride into the fetal heart [20]. In cases in which the first method failed, a second attempt was made using a second method.

In the misoprostol-only group, patients received 400 mcg of misoprostol buccally after the confirmation of no fetal heartbeat on US. Subsequent buccal doses of 400 mcg misoprostol were administered at 4-h intervals until fetal expulsion, or a maximum of 5 doses was reached. For patients with a history of CD, 400 mcg misoprostol buccally was administered at 6-h intervals. In cases where fetal expulsion did not occur after the administration of 5 doses of misoprostol, an alternative regimen involving another set of 5 doses was initiated.

Guided by evidence suggesting the efficacy of mifepristone in second-trimester abortions [1, 2], our local protocol was revised in 2018. We began incorporating mifepristone as a pretreatment to the misoprostol regimen for medication abortion up to 30 + 0/7 weeks’ gestation for all patients. Hence, patients admitted after the protocol changed also received 200 mg of mifepristone orally on the day of feticide. Patients in this group were admitted on the day of feticide and mifepristone administration, which occurred around the same time. On the subsequent day, about 24 h later, a first dose of 800 mcg of misoprostol via the buccal route was administered. The remainder of the protocol was consistent across both groups (400 mcg of misoprostol buccally every 4–6 h until fetal expulsion).

All patients admitted during 2018 after the protocol changed received the mifepristone-misoprostol protocol. Successful medication abortion was defined as fetal expulsion. The abortion interval was measured in hours from the first misoprostol dose in both groups, while hospitalization duration was measured in days, beginning at admission.

After fetal expulsion, additional procedural interventions for retained placental tissue were implements based on visual inspection and bedside US. In cases where the placenta appeared intact and the endometrium measured less than 15 mm on US, surgical aspiration was omitted. In other cases (endometrium measured > 15 mm, when the placenta was not expelled spontaneously within 30 min, or earlier when accompanied by major bleeding), uterine evacuation was performed by manual removal or surgical evacuation using guided US aspiration and curettage.

### Outcomes

The primary study outcome was the duration of abortion, measured as the interval from first misoprostol dose to fetal expulsion. Secondary outcomes were length of hospital stay, need for additional procedural intervention following fetal expulsion and complications. Early complications were defined as postpartum hemorrhage (blood volume > 500 mL), disseminated intravascular coagulation, transfusion of blood products, and early infection (documented temperature > 38.0 °C or use of antibiotics). Late complications were defined as readmission or late infection (defined as infection within the first 30 days after discharge).

### Data

Data were abstracted manually from electronic medical records of all patients at ≥ 20 weeks’ gestation who were admitted for medication abortion from January 2014 to May 2022 and included patient demographics, obstetric and gynecologic history, and information regarding the relevant pregnancy. Parameters related to the abortion procedure, medical regimen, surgical intervention, and complications were also recorded. Duration of hospital stay was calculated beginning at admission.

For study purposes, pre-abortion data, medical abortion duration, and patients’ outcomes were compared between patients who used the mifepristone-misoprostol protocol to the misoprostol-only group.

### Ethical considerations

This study was performed in accordance with the principles of the Declaration of Helsinki. All methods were carried out in accordance with relevant guidelines and regulations. The study protocol was approved by Meir Medical Center Ethics Committee #MMC238-22. Due to the retrospective nature of the study, the Ethics committee waived need for informed consent.

### Statistical analysis

Categorical variables were summarized as numbers and percents. The distribution of continuous variables was evaluated using the Kolmogorov–Smirnov test. For reader ease, all continuous variables were reported as mean and standard deviation, unless mentioned otherwise. The categorical variables were compared between the two groups using chi-square test or Fisher’s exact test. Continuous and ordinal variables were compared between groups using independent samples *t*-test or Mann–Whitney test.

Kaplan–Meier curve was used to describe fetal expulsion time from first misoprostol dose and log-rank test was used to compare between the two groups. Univariate Cox regression was used to study the association between each variable and the time to fetal expulsion. Multivariable Cox regression was used to study the association between mifepristone and the time to fetal expulsion after the first dose of misoprostol. Previous abortions, GA, previous vaginal delivery, and epidural analgesia were considered possible confounders. Multivariable logistic regression was used to study the association between the treatment with mifepristone and the need for procedural interventions, while controlling for potential confounders. Patient age, GA, previous CD, and previous dilation and curettage were considered possible confounders, and a backward method was applied using the Wald test as criteria for variable removal (P 0–1). All statistical tests were two sided and *p* < 0.05 was considered statistically significant. NCSS 2022 software was used for all statistical analyses (NCSS Statistical Software, LLC. Kaysville, UT, USA).

## Results

**Fig. 1:**
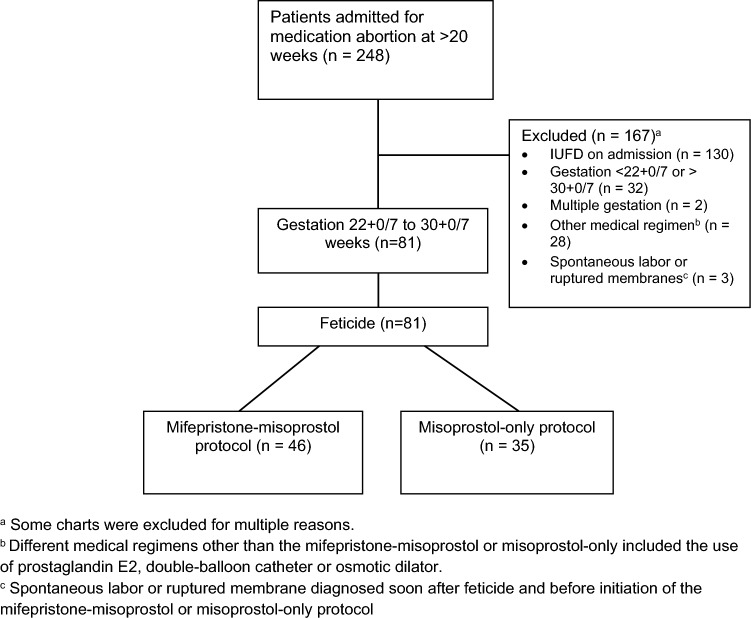
Reviewed charts of patients admitted for medication abortion at > 20 weeks’ gestation from January 2014 to May 2022

During the study period, 248 patients were admitted to our unit for medication abortion at > 20 weeks’ gestation. After excluding patients with multiple gestation, IUFD, treatment with other regimens or protocols, and spontaneous rupture of the membranes or labor, 81 patients met the inclusion criteria: 46 (56%) in the mifepristone-misoprostol group and 35 (44%) in the misoprostol-only group (Fig. 1).

Table 1 presents the demographic and clinical characteristics of the study groups. The mean GA in the mifepristone-misoprostol group (23.8 ± 1.7 weeks) was younger than in the misoprostol-only group (25.4 ± 2.4 weeks, respectively; *p* = 0.002). There were no significant differences in other characteristics between groups.

**Table 1:** Demographic and clinical characteristics of the study groups

Variable^a^	Mifepristone + Misoprostol *n* = 46	Misoprostol-only *n* = 35	*p* value^a^
Age, years	33.5 ± 5.8	33 ± 6.1	0.708
Smoking	4 (8.7%)	1 (2.9%)	0.38
Gravidity	3.1 ± 1.7	2.6 ± 1.5	0.202
Parity	1.4 ± 1.5	1.0 ± 1.1	0.098
Nulliparous	11 (23.9%)	13 (37.1%)	0.196
Previous vaginal delivery	28 (60.9%)	20 (57.1%)	0.735
Previous cesarean delivery	9 (19.6%)	4 (11.4%)	0.37
Previous abortion	23 (50%)	10 (28.6%)	0.052
Previous dilation and curettage	12 (26.1%)	4 (11.43%)	0.16
Gestational age, weeks (mean)	23.8 ± 1.7	25.37 ± 2.4	0.002
22–24	34 (73.9%)	13 (37.7%)	
25–27	10 (21.7%)	17 (48.5%)	
28–30	2 (4.3%)	5 (14.3%)	
Hemoglobin on admission, g/dL	11.6 ± 1.1	11.1 ± 1.3	0.063
Total misoprostol dose, mcg	1608 ± 523	1611 ± 766	0.83
Epidural analgesia	41 (89.1%)	30 (85.7%)	0.74

^a^Data are shown as number (%) or mean ± standard deviation, as appropriate

^b^*P* values derived from *t*-test, Mann–Whitney, chi-square, and Fisher’s exact tests

**Fig. 2:**
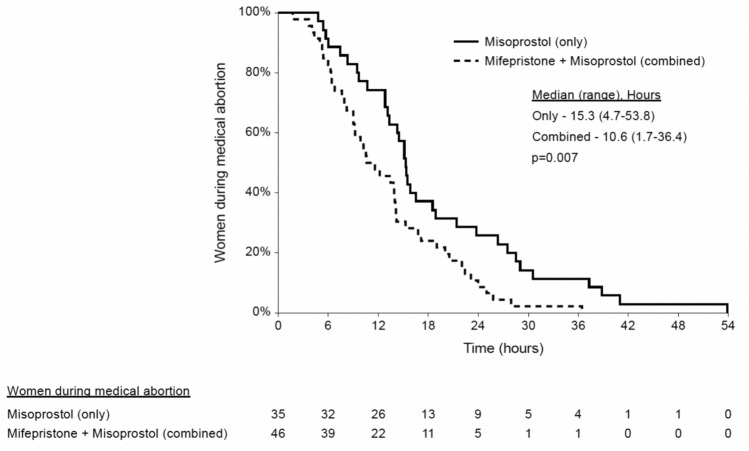
Hours from misoprostol initiation to fetal expulsion during medication abortion at ≥ 22 + 0/7 to < 30 + 0/7 weeks’ gestation

All patients delivered vaginally except one in the misoprostol-only group who required cesarean hysterotomy (due to patient request after failed fetal expulsion following 5 doses of misoprostol). In the mifepristone-misoprostol group, the mean interval from mifepristone administration to the first dose of misoprostol was 20.8 ± 4.1 h. The interval until fetal expulsion was shorter in the mifepristone-misoprostol group [median (range): 10.6 (1.7–36.4) hours vs. 15.3 (4.7–53.9), *p* = 0.007; Fig. 2]. In addition, while fetal expulsion occurred in most patients in the mifepristone group within 24 h after the first misoprostol dose, in the misoprostol-only group about 30% had not completed the procedure by 24 h. This finding was significant after controlling for previous abortions, GA, previous vaginal delivery, and epidural analgesia, using multivariable Cox regression [odds ratio (OR) 1.7, 95% confidence interval (CI) 1.03–2.9, *p* = 0.03].

Table 2 presents the outcomes of the study groups. Patients in the mifepristone-misoprostol group had shorter hospital stays (3.5 ± 1.1 vs. 4.1 ± 1.2 days, respectively; *p* = 0.013). This group had higher rate of procedural interventions for retained placental tissue (60.9% vs. 34.3%, *p* = 0.018). However, a multivariable logistic regression revealed that mifepristone was not independently associated with increased need for procedural intervention. Older GA was associated with fewer procedural intervention, while previous dilation and curettage was associated with increased need for procedural intervention (Table 3). There were no other significant differences in terms of early or late complications.

**Table 2:** Outcome characteristics of study groups

Variable^a^	Mifepristone-misoprostol *n* = 46	Misoprostol-only *n* = 35	*p* value^f^
Procedural intervention^b^	28 (60.9%)	12 (34.3%)	0.018
Length of hospital stay (days)	3.5 ± 1.1	4.1 ± 1.2	0.013
Hemoglobin after fetal expulsion (g/dL)	10.9 ± 1.6	10.2 ± 1.5	0.13
Total early complications^c^	9 (19.6%)	4 (11.4%)	0.37
Postpartum hemorrhage	2 (4.4%)	3 (8.6%)	0.65
Disseminated intravascular coagulation	1 (2.2%)	0	> 0.99
Transfusion of blood products	2 (4.3%)	0	0.5
Infection^d^	8 (17.4%)	1 (2.9%)	0.07
Late complications^e^	3 (6.5%)	0	0.25
Readmission	2 (4.4%)	0	0.5
Late infection	3 (6.5	0	0.25

^a^Data are shown as number (%) or mean ± standard deviation, as appropriate

^b^Surgical or manual removal of placental tissue after fetal expulsion

^c^Total complications include PPH, DIC, blood product transfusion, and infection

^d^Documented temperature > 38.0 °C or use of antibiotics

^e^Late complication includes readmission within 30 days after hospital discharge and late infection (documented temperature > 38.0 °C or use of antibiotics after hospital discharge)

^f^*p* values derived from *t*-test, Mann–Whitney, chi-square, and Fisher’s exact tests

**Table 3:** Factors associated with increased procedural interventions following fetal expulsion^a^

Variable	Odds Ratio	95% CI	*p* value
Gestational age	0.73	0.56–0.94	0.01
Previous cesarean delivery	3.29	0.81–13.3	0.09
Mifepristone	1.56	0.54–4.46	0.4
Previous dilation and curettage	4.84	1.17–20.05	0.03

^a^Procedural interventions: manual removal of placenta, uterine revision, and aspiration and curettage

## Discussion

The results of this retrospective cohort study indicate that pretreatment with mifepristone before administering misoprostol for medication abortion at GA of 22 + 0/7 to 30 + 0/7 weeks was associated with shorter abortion duration. The median time for fetal expulsion was 10.6 h, significantly shorter than 15.3 h in the misoprostol-only group. In addition, a larger proportion of patients in the misoprostol-only group had abortion time longer than 24 h. Patients in the mifepristone-misoprostol group required more procedural intervention after fetal expulsion. No notable increase in other complications was associated with the use of mifepristone.

The current study successfully demonstrates the potential effectiveness of mifepristone on decreasing abortion duration in this specific population of patients at 22 + 0/7 to 30 + 0/7 weeks of gestation. We included cases in which medication abortion was initiated after feticide for uniformity and because medication abortion after IUFD might be shorter, regardless of the regimen used [2, 9, 21–23]. Patients with GA ≥ 22 + 0/7 weeks were included in this study because of the sparse data available at this GA range, and because feticide is performed at our department at this GA. Medication abortion using mifepristone-misoprostol or misoprostol-only was limited to GA of < 30 + 0/7 weeks in accordance with the local protocol, patients at later GA were managed with different regimens [2, 23].

Although previous studies established the efficacy of mifepristone for decreasing the duration of medication abortion in the second trimester, the available data at > 22 + 0/7 weeks are limited [2, 9, 23]. Previous studies investigated mifepristone efficacy in second-trimester abortions, consistently indicating shorter labor duration [5, 9, 24]. However, these studies included pregnancies with GA beginning from 12 weeks. One study, that included pregnancies at 20–27 weeks, found the duration was shorter with the addition of mifepristone. However, it included pregnancies with IUFD along with TOP in the same group [12]. In contrast, another study that included only patients who underwent TOP at ≥ 24 + 0/7 weeks found no difference in time to fetal expulsion with the use of mifepristone [11]. However, the protocol in that study included the insertion of osmotic dilators in all patients before the first dose of misoprostol.

The present study showed that mifepristone was also associated with a minimal decrease in overall hospital stay, as seen in previous studies. Since the shorter median time until fetal expulsion in the mifepristone-misoprostol group was about 5 h shorter and the mean hospital stay in the mifepristone group was 3.5 ± 1.1 days vs. 4.1 ± 1.2 days in the misoprostol-only group (*p* = 0.013), other factors than mifepristone alone might have contributed to the difference. The retrospective nature of the study makes it difficult to assess these factors but it is reasonable to assume that at least, mifepristone did not prolong hospital stay.

Patients in the mifepristone-misoprostol group had a higher rate of procedural interventions after fetal expulsion (60.9% vs. 34.3%, respectively; *p* = 0.018). Although mifepristone was not found to be independently associated with retained placental tissue (Table 3), as shown in previous studies, this group might have experienced a higher intervention rate due to the difference in the mean GA [3, 6, 11, 12]. Another possible explanation for this difference is a lower threshold for intervention in recent years, when the mifepristone-misoprostol protocol was used. However, and despite the retrospective nature of this study, to the best of our knowledge the main change in the treatment protocol during the study period was the addition of mifepristone, as described earlier.

The current study had several notable strengths. The cohort was exclusively composed of cases where medication abortion carried after feticide at a GA of ≥ 22 + 0/7 to < 30 + 0/7 weeks, while most other studies lack data after 22 + 0/7 weeks. Second, both study groups underwent treatment within the confines of the same tertiary, university-affiliated medical center, following similar protocols, with the main difference being the addition of mifepristone as pretreatment.

This study had certain limitations. The relatively modest sample size, coupled with its retrospective nature, bears the potential of introducing undetected biases and constrains the applicability of the findings on a broader scale. Notably, there was a disparity in the mean GA between the groups. This difference is mostly notable in the later GA (25 to 27 weeks and in > 28 weeks), a subgroup of patients with especially limited data in the current literature. A plausible explanation for this difference might be earlier diagnosis of genetic abnormalities prompting TOP in recent years, when the mifepristone-misoprostol regimen was used, as a result of the increased use of chromosomal microarray analysis for prenatal diagnosis in our local population. However, a univariate Cox regression analysis revealed no discernible correlation between GA and the interval until fetal expulsion. Moreover, a multivariate Cox regression indicated that mifepristone was independently linked to a shorter interval until fetal expulsion. The difference in the first dose of misoprostol between the groups (800 mcg in the mifepristone-misoprostol group, compared with 400 mcg in the misoprostol-only group) was another limitation. However, there was no difference in the cumulative dosage of misoprostol administered between the groups (1608 ± 523 vs. 1611 ± 766 mcg, *p* = 0.83).

## Conclusions

This study has successfully established an association between pretreatment with mifepristone and shorter interval to fetal expulsion among patients who underwent medication abortion at GAs 22 + 0/7 to 30 + 0/7 weeks, without increasing length of hospital stay or complication rates. This contribution is particularly significant considering the paucity of data within this specific subject area and could potentially provide valuable insights to both patients and clinicians when selecting the optimal treatment regimen. However, a RCT is needed to comprehensively explore the impact of mifepristone within this specific GA.

## Data Availability

The data used in this study are available upon reasonable request from the corresponding author.
